# Autistic Children Show a Surprising Relationship between Global Visual Perception, Non-Verbal Intelligence and Visual Parvocellular Function, Not Seen in Typically Developing Children

**DOI:** 10.3389/fnhum.2017.00239

**Published:** 2017-05-11

**Authors:** Alyse C. Brown, David P. Crewther

**Affiliations:** Centre for Human Psychopharmacology, Swinburne University of TechnologyMelbourne, VIC, Australia

**Keywords:** Autism Spectrum Disorders, perception, parvocellular, magnocellular, inspection time (IT), non-verbal intelligence, visual evoked potential, neural efficiency

## Abstract

Despite much current research into the visual processing style of individuals with Autism Spectrum Disorder (ASD), understanding of the neural mechanisms is lagging, especially with respect to the contributions of the overlapping dichotomies of magnocellular/parvocellular (afferent neural pathways), global/local (perception) and dorsal/ventral (cortical streams). Here, we addressed this deficiency by measuring inspection times (ITs) for novel global/local stimuli as well as recording nonlinear visually evoked potentials (VEPs), in particular, magnocellular and parvocellular temporal efficiencies. The study was conducted on a group of male ASD children and a typically developing (TD) group matched for mean age and mean non-verbal intelligence, as measured by the Raven’s Progressive Matrices. The IT results did not differ between groups, however a negative correlation between global IT and Raven’s score was found in the ASD group, that was not evident in the TD group. Nonlinear VEP showed the ASD group had smaller amplitude parvocellular-generated second order responses compared to the TD group. This is a sign of improved temporal responsiveness in ASD vs. TD groups. Principal Component Analysis linked global IT, non-verbal intelligence scores and VEP parvocellular efficiency in a single factor for the ASD but not the TD group. The results are suggestive of a constraint on pathways available for cognitive response in the ASD group, with temporal processing for those with ASD becoming more reliant on the parvocellular pathway.

## Introduction

Autism Spectrum Disorder (ASD) encompasses the previously discrete diagnoses of autism, Asperger’s disorder and pervasive developmental disorder (PDD) not otherwise specified (NOS; American Psychiatric Association, 2013). Its features include social deficits and communication difficulties, stereotyped or repetitive behaviors and interests, sensory issues, and in many cases, cognitive delays. Visual perception in ASD is characterized by an atypical bias towards local perception, thought to often override the normal global precedence for objects (Dakin and Frith, 2005). Indeed, Simmons et al. (2009), in review, proposed that unusual sensory processing could be causal in ASD symptomatology.

Two cognitive theories have emerged to explain anomalous ASD perception. The enhanced perceptual functioning (EPF) theory suggested that there is an over-development of low-level perceptual operations that causes detection, primary discrimination and other low-level abilities to be enhanced (Mottron et al., 2006). By contrast, the Weak Central Coherence (WCC) theory proposed that individuals with ASD have a local processing style bias as they use gestalt principles less (Happé, 1999; Happé and Frith, 2006). Both of these theories allude to there being less processing in the later stages of visual processing. A meta-analysis of global/local perception in ASD by Van der Hallen et al. (2015) combining data from several different tasks, found no evidence of enhanced local processing, and found that global processing was slowed. However, a meta-analysis of global/local processing by Muth et al. (2014) in which the tasks were separately analyzed showed that enhanced local processing in ASD was not a general finding but was task-dependent. In terms of theories based on neural processing, the dorsal stream vulnerability hypothesis (Braddick et al., 2003) posited that global form and motion sensitivity is particularly susceptible to damage in many neurodevelopmental disorders including autism, because of the more stringent neural temporal requirements of the magnocellular pathway that supports these abilities. Recently, the idea of altered neural noise has been used to explain aspects of autism (Simmons et al., 2009; Pellicano and Burr, 2012; Greenaway et al., 2013), however work is still required to make a strongly predictive theory.

The first measures of global/local precedence used the Navon figures task (Navon, 1977) where participants are asked to respond to the large letters (global form) or small letters (local form) that make up the large letter. In the years since the emergence of Navon figures, the understanding of global and local perception has become more sophisticated. Thus, the notions of edge vs. object, of part vs. whole, have been summarized according to grouping principles based on proximity; good continuation; similarity; closure; symmetry and parallelism; and convexity (Wagemans et al., 2012). Now there is a growing interest in identifying the neural mechanisms at play. An outstanding example of differential neural analysis of global vs. local perception is the diamond illusion, where percept fluctuates between four ungrouped moving lines or the lines appearing to move coherently as a diamond shape. Functional MRI activations (Fang et al., 2008), show the local percept activating primary visual cortex (V1) and the global form percept predominantly activating Lateral Occipital Complex (LOC), at the expense of V1 activation.

**Figure 1:**
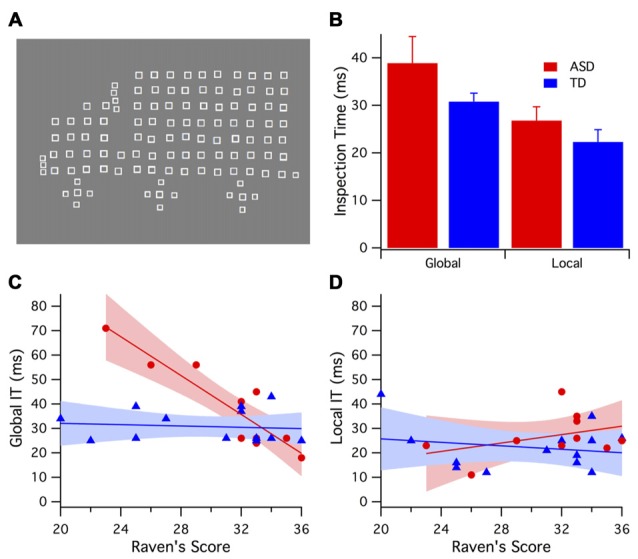
**(A)** An example of global/local stimuli presented during the inspection times (ITs) tasks. **(B)** Mean and error bars (1 SE) of global and local IT for autism spectrum disorder (ASD; red) and typically developing (TD; blue) groups. The TD group showed consistently lower thresholds. **(C)** Global ITs demonstrated a negative relationship with Ravens score for the ASD group (red), whereas the TD group (blue) global IT showed no significant correlation with Raven’s score. **(D)** Local IT showed no significant correlation with Ravens score for either ASD (red) or TD (blue) groups.

While the classic global/local Navon figures task is ubiquitous, we argued that it is not the optimal stimulus for determining global/local neural processing differences. This is on the basis that both global and local outcomes of task demand require recognition of a letter, and as such, the likely activation site—the neural end-point of recognition (Grill-Spector et al., 2004), is the same—the visual word-form area (Lux et al., 2004; Billington et al., 2008), no matter if a global or local letter is identified. Similarly, hierarchical figures, based on the same Navon principles but using shapes as elements (Mottron and Belleville, 1993; Plaisted et al., 1999; Rinehart et al., 2000; Bölte et al., 2007) again fail to address the similarity of global/local brain activations (Mottron et al., 2006). We addressed this problem by increasing the comparative complexity between the global and local stimulus levels to ensure that the global images are processed further into the inferotemporal pipe-line than the local images. The novel stimuli developed were complex forms made up of simple forms (e.g., truck/squares, fish/circles, as shown in Figure 1A).

The second part of this study aimed to examine the tenuous link between dorsal stream vulnerability and poor global form processing in ASD that has been suggested by Braddick et al. (2003). Central to Braddick’s argument is that the dorsal stream is made vulnerable due to the stringent neural timing requirements of the magnocellular pathway that dominates its input (Milner and Goodale, 2008). In the lateral geniculate nucleus (LGN) the magnocellular neurons have a latency advantage over the parvocellular neurons because of faster axonal conduction speeds (Maunsell and Gibson, 1992; Maunsell et al., 1999). Physiological estimates of the magnocellular advantage (Laycock et al., 2007) in activating human cortical area V1, range from 25 ms to 30 ms (Baseler and Sutter, 1997; Klistorner et al., 1997; Sutherland and Crewther, 2010; Jackson et al., 2013). Possessing high temporal resolution, visual evoked potential (VEP) studies of the ASD processing style have shown abnormal responses in children (Vandenbroucke et al., 2008; Pei et al., 2009; Fujita et al., 2011) and adults (Jemel et al., 2010) with ASD. Interestingly, differences in nonlinear VEP responses between groups high and low in autistic tendency have been attributed to altered magnocellular function (Sutherland and Crewther, 2010; Jackson et al., 2013).

Nonlinear VEP allows for the analysis of the temporal structure of responses that occur during rapid stimulation. The first order response is similar to the impulse response function of a standard VEP, while the second order responses measure the effect of prior stimulation as a function of interaction time. This temporal analysis allows for an independent analysis of the magnocellular and parvocellular pathways by capitalizing on their different contrast and temporal response properties in the second order response (Klistorner et al., 1997). Previous research has used nonlinear VEP results as an index of neural efficiency with amplitude reduction in the second order response associated with more efficient response recovery (Bauer et al., 2011; Jackson et al., 2013). In neurotypicals with high autistic tendency, nonlinear VEP recordings have revealed magnocellularly-generated second order nonlinearities are less efficient (greater magnitude) compared to those low in autistic tendency (Jackson et al., 2013).

### Intelligence and Perception

While ASD has been recognized as being associated with generally lower IQ, the nature of the intellectual disability in ASD is not classically subnormal (Frith, 2003; Brown et al., 2017). This understanding is based on the profile of subtest scores that individuals with ASD receive on the Wechsler Intelligence Scale for Children (WISC), for example, high performance on the Block Design task even as general IQ decreases (Lincoln et al., 1988; Allen et al., 1991; Shah and Frith, 1993; Happé, 1994). In addition, the language-based subtest scores that are predictive of general IQ in a typical population are not predictive in ASD (Bello et al., 2008; Charman et al., 2011; Courchesne et al., 2015). One of the fundamental tenets of general intelligence—speed of processing, appears not to contribute to low intelligence for those with ASD (Anderson, 2008). A review by Brown et al. (2017) extends these deviations from the normal relation between ASD and IQ to visual processing. For example inspection time (IT) has a well-studied relationship with intelligence in typically developing (TD) individuals, established since the late 1970’s (Nettelbeck, 1982). Meta-analysis of the standard IT task (tuning fork with unequal legs) shows a mean correlation between IT and IQ of −0.54 in normal healthy adults (Kranzler and Jensen, 1989). However, for ASD populations, correlations appears variable (Scheuffgen et al., 2000; Wallace et al., 2009; Barbeau et al., 2013), depending on the type of intelligence assessment and the inclusion or not of Asperger subtype (DSM-IV) within the autistic sample.

Hence, the current study aimed to investigate neurophysiological function and visual processing speed in relation to non-verbal intelligence in groups of male ASD and TD school-age children matched in mean age and non-verbal intelligence. We measured IT for novel global/local figures differentiated by level of complexity, hypothesizing that the ASD group would show shorter IT for the local condition and longer IT for the global condition compared to the TD group. In addition, we recorded multifocal VEP to test whether differences in magnocellular function found in adult high vs. low autistic tendency (Jackson et al., 2013) extended to our clinical child sample. Thus in this design, measures related to the three dichotomies: speed of global/local perception, non-verbal IQ and physiological measures of magnocellular/parvocellular function were compared in the same populations.

## Materials and Methods

### Participants

Participants were recruited from a primary school in the eastern suburbs of Melbourne, Australia. The ASD group comprised of male children aged 7–11 years. Due to ethical restrictions tied to this study, the ASD diagnostic status was confirmed via the school’s special needs program and departmental evaluation of diagnostic records. We requested to be given names only of children with a full clinical diagnosis that included both a pediatrician and a psychologist. All members of the ASD group met the special needs criteria on the basis of autism (in this case assessed under DSM-IV). The TD group was matched for chronological age, gender and non-verbal intelligence using the Raven’s Colored Progressive Matrices test (RCPM; Raven et al., 1998)—see Table 1.

**Table 1:** **Comparison of Autism Spectrum Disorder (ASD) and typically developing (TD) groups**.

	Age (years)		Raven’s score		Child CBPQ	
	*M*	*SD*	*M*	*SD*	*M*	*SD*
ASD (*n* = 11)	9.09	1.30	29.73	6.21	90.91	25.71
TD (*n* = 14)	8.79	1.31	29.86	5.07	45.36	23.08

The study was approved by the university’s human research ethics committee as well as by the Department of Education and Early Childhood Development of Victoria. As the participants were under the age of 18 years consent was obtained from a parent or legal guardian of the participant. Participants were asked at the beginning of each new task if they would like to participate in the activity. If the child said no or became distressed during the testing session testing was stopped. Written informed consent was obtained from parents of children prior to collection of data. Severity of autistic symptoms was measured using the Cambridge University Behavior and Personality Questionnaire for Children (CBPQ-Child; Auyeung et al., 2008) which the parents of both child participant groups were required to complete at the start of the study. Exclusion criteria existed for individuals diagnosed with epilepsy or abnormal vision (other than refractive error). On the CBPQ, two TD children scored higher than 76 (/150)—the threshold score thought to separate clinical from healthy controls with 95% confidence (Auyeung et al., 2008), but these individuals were retained in the TD group so as to not skew the distribution of data.

### Procedure

First, participants completed the Ravens Colored Progressive Matrices test (RCPM) which took approximately 10 min to complete, followed by a computer based global/local IT task, created using VPixx software^1^. Testing was based around two periods with a 2-week gap in between. The first period acted as a familiarization to the tasks and the testing protocols. The second period included both psychophysical and electrophysiological (VEP) data collection.

### IT Using Global/Local Figures

IT refers to the exposure duration required for a participant to reliably identify a stimulus. The global/local stimuli used in the IT tasks consisted of complex figures (fish, trucks and butterflies) each made up of simple geometrical shapes (circles, triangles or squares)—for an example of the stimuli see Figure 1A. Global stimuli were displayed in two different orientations using left/right reversal to decrease the possible reliance on local feature detection during the global condition. The number of local elements in each global stimulus (truck, fish, butterfly) was controlled, differing by no more than 5. The dimensions of the global images displayed were 32°W × 25°H, with the small local shapes 1°W × 1°H. Images were created using Mathematica (Wolfram Research).

The children were first familiarized with the stimuli; the experimenter asked “what do you see” for each image, with the objective of making sure the children were able to correctly identify the stimuli in either the global or local aspect. The IT task was run twice each time with a different instruction; “What picture did you see?” (global IT) or “What shape did you see?” (local IT). The order of global and local recognition tasks was counterbalanced across subjects. Depending on the participants’ capabilities (verbal or non-verbal) participants gave their responses verbally or by pointing to one of the three images on a print-out of the stimuli, the experimenter then entering the participants’ responses into the program. Participants were instructed to guess, if they were unsure. Before starting each IT task, 10 practice trials were given so to allow for adaptation to the new instructions. Each IT task consisted of 35 trials, with threshold reached in approximately 3 min. A three-alternate forced-choice (3AFC) paradigm was used and stimulus presentation was fully randomized. Each trial started with a fixation cross displayed for 0.5 s followed by the presentation of one of the three possible target stimuli which was displayed for a varying amount of time followed by a masking stimulus of dynamic random dot noise stimulus for 17 ms. The task waited for a button press response before moving on to the next trial. Target stimulus display time (starting at 2 s yielding correct percept in all participants) was modulated under a PEST procedure (built into VPixx) the threshold estimate used as the participants’ inspection time.

### Nonlinear VEP

Gold cup electrodes filled with electrode paste were placed at prepared sites Oz referenced to Fz (10/20 system) with the right ear used as ground. During the recording of the multifocal stimulus, participants were directed to maintain fixation on the red dot in the middle of the visual stimulus. Throughout the recordings, one experimenter watched the participant’s eye gaze and when necessary, reminded the participant to look at the red central dot. A second experimenter watched the waveforms for evidence of electromyographic noise and reminded the participant to relax if the recording started to get noisy.

To record the nonlinear VEP, a software/hardware combination was used, with the binary pseudorandom multifocal stimulus (9 square patches) coded in VPixx and employing a DATAPixx^1^ interface box for strict video frame registration. Each of the 9 square patches fluctuated between two luminance levels under the control of a pseudo-random *m* = 14 binary sequence with each patch set to an independent flicker sequence. To an observer these stimuli appear to rapidly flicker. Two temporal stimulus contrasts were used—24% and 96% contrast. The 4 min pseudo-random binary sequence was divided into 1-min segments of recording to allow for rest breaks (a few second, during which time the participant was asked to blink rapidly three or four times to hydrate their corneas). The stimulus was presented using a 75 Hz frame rate CRT monitor. The central square patch stimulus (8° × 8°) was larger than those used in previous multifocal VEP studies (Klistorner et al., 1997) to better accommodate deviations from fixation.

### Data Pre-Processing

The signal was amplified 50,000 times with band-pass filtering between 1 Hz and 1 kHz. Data was sampled at 1 kHz. Only responses recorded from the central patch were analyzed. Using the Wiener kernel expansion (for a detailed explanation, see Sutter, 2000; Jackson et al., 2013) first order Kernel, K1 and second order Kernels K2.1 and K2.2 of the VEP were extracted using custom software (Lab VIEW, National Instruments). For example, the first order response (K1) for a pseudo-random sequence of black (b) and white (w) stimuli is the average of all responses to white stimuli Rw, minus the average of responses to the black stimuli Rb during the pseudorandom sequence, i.e., K1 = 1/2 (Rw − Rb). The second order responses take account of the stimulation history. The first slice of the second order response (K2.1) takes into account stimulation one frame back so it represents a comparison between two consecutive frames when a transition has happened and when a transition has not occurred, i.e., 1/4(Rww + Rbb − Rwb − Rbw). The second slice (K2.2) takes into account the same responses but with an additional intervening frame of both polarities. The difference in temporal recovery responses in first slice (13 ms with a 75 Hz frame rate) and second slice (27 ms) of the second order kernel separates inputs of the magno and parvocellular pathways (Klistorner et al., 1997; Jackson et al., 2013) on the basis of contrast gain, saturation and peak latency (Baseler and Sutter, 1997; Klistorner et al., 1997; Crewther et al., 1999; Laycock et al., 2007; Sutherland and Crewther, 2010; Jackson et al., 2013).

Mean average waves were calculated for each group. One-way between-group ANOVAs were used to examine group differences in the amplitudes and latencies of all peaks. To reduce between-subject variation in recording conditions (e.g., skull thickness), ratios of the second order to first order amplitudes of the most prominent parvocellular and magnocellular generated peaks (Klistorner et al., 1997; Jackson et al., 2013) were calculated for each participant. The parvocellular ratio was defined by the ratio of peak to peak amplitudes K2.2:N90-P130/K1:N90-P130 while the magnocellular ratio was similarly defined by the ratio K2.1: N60-P90/K1: N60-P90. Through this definition, the smaller the ratio (relatively less second order than first order response), the higher the neural efficiency demonstrated by the pathway producing that peak.

## Results

In all the tasks there was one consistent outlier (from the ASD group); this participant was deleted from the data set as their attention and comprehension levels were too low to complete the tasks. Further outlier checks were performed for individual tasks. Following outlier removal, comparison of Raven’s scores showed no significant difference between groups (one-way analysis, *t* = 0.44, *p* = 0.66). Comparison of scores on the Child CBPQ test—a measure of autistic characteristics, showed a clear difference between the ASD and TD groups (one-way analysis, *t* = 3.22, *p* = 0.006) with the ASD group scoring higher on autistic characteristics than the control group.

### Global/Local IT

When familiarizing the children with the stimuli, 3 out of the 11 ASD children identified the local shapes in the stimuli before the global image; there were no instances of this occurring in the TD group.

A two-way mixed ANOVA was conducted with participant sample (TD vs. ASD) as a between-groups variable and IT (global vs. local) as a within-groups variable. The local IT was shorter than the global IT time for both groups *F*_(1,21)_ = 9.235, *p* < 0.006. The TD group had consistently shorter mean ITs compared with the ASD group (Figure 1B), however between-group differences were not significant *F*_(1,21)_ = 4.03, *p* = 0.058, and there was no significant interaction *F*_(1,21)_ = 0.384, *p* = 0.542.

Further analysis of the IT data found a negative relationship between Raven’s scores and global IT (see Figure 1C). A one-tailed correlation analysis controlling for the variable age, revealed that Raven’s score explains 83% of the variance in ASD global IT: *F*_(1,8)_ = 38.72, *p* < 0.001. However, little correlation was evident between Raven’s score and global IT for the TD group *F*_(1,9)_ = 0.727, *p* < 0.416. A permutation test revealed that the between-groups correlations for Raven’s score and global IT were significantly different (*p* < 0.001). Remarkably, there were no significant correlations observed between local IT and Raven’s score for either the ASD *F*_(1,8)_ = 1.00, *p* = 0.350 or TD *F*_(1,9)_ = 0.087, *p* = 0.775 groups (see Figure 1D).

### Nonlinear VEP

**Figure 2:**
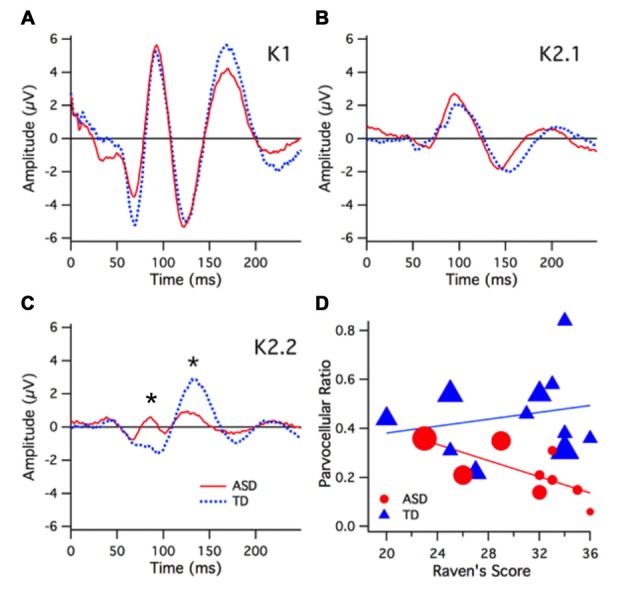
**High (96%) contrast nonlinear visually evoked potentials (VEPs) group kernel responses and interactions**. Color key for groups; ASD (red) and TD (blue). In these graphs depicting VEP group average responses the positive (P) peaks are in the up direction and negative (N) peaks are in the down direction. **(A)** The earliest first order kernel cortical activity (N60) shows a smaller mean amplitude for ASD vs. TD (not significant). **(B)** The second order kernel first slice (K2.1) was not different between ASD and TD groups. **(C)** The second order, 2nd slice nonlinearity (K2.2) was significantly smaller in amplitude for the ASD group compared with TD at P1 (90 ms) peak (*p* < 0.006) and P2 (130 ms) peak (*p* < 0.05) and as indicated by asterisk. **(D)** Parvocellular nonlinearity ratio (estimated for the major P130 peak) diminishes as a function of Raven’s score for the ASD but not TD group. The marker size here represents global IT. A three-way relationship is evident in the diminishing marker size (shorter IT) as Raven’s score increases for the ASD but not the TD group.

The largest group differences were seen in the K2.2 kernel response to the high contrast (96%) stimulus (see Figure 2C). At high contrast, the K2.2 P1 peak amplitude (90 ms) was smaller for the ASD group *F*_(1,17)_ = 9.88, *p* < 0.006 as was the P2 peak amplitude (130 ms) *F*_(1,17)_ = 5.63, *p* < 0.029 compared to the TD group. No further group peak amplitude differences were found in any of the other kernels in response to the high contrast stimulus.

At low contrast (24%), there were no significant differences between the groups’ mean average peak amplitudes for all of the kernel responses (K1, K2.1, K2.2). At low contrast, group differences in latency were found. In the K2.1 kernel slices, the ASD group showed a shorter latency in the N1 (60 ms) peak *F*_(1,17)_ = 6.64, *p* < 0.02 and a longer P2 (130 ms) latency peak of the *F*_(1,17)_ = 6.45, *p* < 0.02 compared to the TD group. These peak latency differences are for peaks that are also noted as being rather platykurtotic and of small amplitude, raising the risk of inclusion of multiple small peaks within the sample window for peak analysis when taken across the two groups of individuals.

A one-way between-groups ANOVA for the high contrast data revealed that the ASD group had significantly smaller parvocellular nonlinearity ratios (greater neural efficiency) compared to the TD group *F*_(1,18)_ = 10.3, *p* < 0.005. No group difference was found for the magnocellular nonlinearity ratio *F*_(1,18)_ = 2.02, *p* = 0.173.

Regression analysis revealed that the Raven’s score explains 48% of the variance in the ASD parvocellular nonlinearity ratio, *F*_(1,7)_ = 6.52, *p* = 0.038, but no significant relationship in the TD group was found. The correlation between parvocellular nonlinearity ratio and global IT (RSq = 0.42, *F* = 4.98, *p* = 0.06) showed a strong trend for the ASD group where there was none for the TDs. The correlations between global IT and parvocellular ratio (ASD: RSq = 0.42, *F* = 5.05, *p* = 0.058; TD: RSq = 0.039, *F* = 0.37, *p* = 0.56), are different (*p* = 0.038) for the two groups, as demonstrated by a permutation test. Figure 2D shows a 3-way interaction plot between Ravens, global IT and parvocellular nonlinearity ratio.

Interestingly, children with a Ravens score ≥30 all showed short global IT values (see Figure 2D), however had different levels of neural efficiency. When only those children with Raven’s score ≥30 were considered, a one-way ANOVA revealed that the ASD sub-group had a smaller parvocellular nonlinearity ratio (higher parvocellular neural efficiency) compared to the TD sub-group *F*_(1,12)_ = 15.64, *p* < 0.002.

### Principal Components Analysis

To investigate relations between all of the variables recorded in this study, a multivariate correlational analysis was conducted on the variables global IT, local IT, parvocellular VEP ratio, magnocellular VEP ratio and Raven’s score, comparing the ASD and TD groups. We used principal components analysis (PCA) to find the major components (eigenvalues >1) for the two groups (see Table 2).

**Table 2:** **Principal components analysis (PCA) analysis for five variables, showing leading eigenvalues, cumulative percentage contribution and probability for ASD and TD groups**.

ASD				TD			
Number	Eigenval	Cum %	Prob > *χ*^2^	Number	Eigenval	Cum %	Prob > *χ*^2^
1	2.643	52.87	0.0004*	1	1.819	36.37	0.352
2	1.237	77.60	0.0079*	2	1.386	64.10	0.439

*Indicates significant results.

Two eigenvectors showed significant contributions to explaining the cumulative variance for the ASD group, however none of the eigenvectors for the TD group showed significance. The first eigenvalue of 2.643 was associated with an eigenvector explaining nearly 53% of the variation in that population data. An inspection of the contribution of the five variables to the leading eigenvectors (see Table 3) shows that the first factor received virtually no contribution from magnocellular function and a relatively small contribution from local IT. However, Raven’s scores, parvocellular VEP ratio and global IT were all strong contributors.

**Table 3:** **Comparison of leading eigenvectors from PCA analysis of five variables (global, local inspection time (IT); magno, parvo visually evoked potential (VEP); non-verbal intelligence)**.

	ASD		TD	
	1	2	1	2
RCPM	−0.595	0.147	0.349	−0.319
Parvo_VEP	0.498	−0.091	0.595	0.390
Magno_VEP	0.033	0.776	0.552	0.418
IT_local	−0.255	−0.601	−0.284	0.521
IT_global	0.576	−0.080	−0.371	0.548

**Figure 3:**
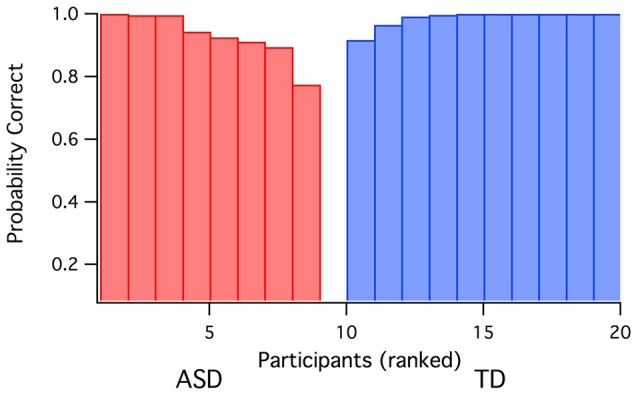
**Prediction probabilities for group membership (ASD vs. TD) based on discriminant analysis using Raven’s score, global IT and parvocellular efficiency data as inputs**. One ASD participant was incorrectly predicted as TD.

Quadratic discriminant analysis was carried out (jmp; SAS corporation) using the three variables (i.e., Raven’s score, global IT and parvocellular efficiency) with a major contribution to the first eigenvector for the ASD. Using a leave-one out validation technique across the whole sample, the model generated only one false prediction of group membership—see Figure 3).

## Discussion

Despite claims of diversity in the genetics of ASD (Happé et al., 2006), the results presented here show a singular relationship linking non-verbal intelligence, global perception and the neural efficiency of the parvocellular pathway, present in the ASD group, but not shown in the TD group.

In this study we used inspection time to measure the rapidity of recognition of the global and local levels of our novel stimuli. These stimuli were designed to probe different depths of the inferotemporal pipeline of cortical areas when attending to the global vs. local level. We found that global IT and Raven’s score were significantly negatively correlated for the ASD but not for the TD group. No significant correlations were found in the local IT condition for either ASD or TD groups. In the conventional IT task (tuning fork with unequal legs) intelligence has an established weak relationship in the typical population (Nettelbeck, 1982). Reports of correlational relationships between TD and standard IT, and the absence of the same relationship in ASD are mixed and seem to be dependent on measures of IQ and type of ASD group used (Scheuffgen et al., 2000; Wallace et al., 2009; Barbeau et al., 2013). We propose that the added complexity in the Global IT condition might boost the IT relationship with IQ in an ASD population.

The literature is supportive of a relationship between visual processing and mental ability in ASD (Brown et al., 2017). The report of correlations between full-scale WAIS scores and performance on biological (Koldewyn et al., 2010; Rutherford and Troje, 2012) and coherent (Jones et al., 2011) motion tasks (associated with ASD populations but not TD groups), appears qualitatively similar. However, the current study shows an ASD relationship dependent on mental ability in a static visual processing task. Furthermore, the results show that this relationship between visual processing and mental ability is not general but is potentially driven by the degree of stimulus complexity. Notably, this study demonstrates the danger of generalizing findings from high functioning ASD populations as representative of all those with ASD. Our study, together with others (Pei et al., 2009; Koldewyn et al., 2010; Jones et al., 2011; Rutherford and Troje, 2012) demonstrates an interaction between ASD and intelligence, not found in the control group.

The comparison of nonlinear VEP between ASD and TD groups did not show differences in magnocellular processing, a difference expected from studies of autistic tendency in high functioning populations (Braddick et al., 2003; Dakin and Frith, 2005; Simmons et al., 2009). However, most neurally based explanations in the literature, targeting magnocellular function as a likely cause of perceptual differences in autism, have generally intuited the neural cause from psychophysical experiments (Dakin and Frith, 2005; Simmons et al., 2009). Articles assessing physiological function in ASD are confined to high functioning ASD populations (Vandenbroucke et al., 2008; Jemel et al., 2010; Schwarzkopf et al., 2014), or TD groups with high or low autistic tendency (Sutherland and Crewther, 2010; Jackson et al., 2013).

In the current study, where non-verbal intelligence was included as a factor, the major group physiological differences revolved around the parvocellularly generated nonlinearities. The parvocellular activation (second order K2.2 response) showed significantly smaller P2 amplitudes for the ASD group at high contrast visual stimulation (see Figure 2) compared to the TD group.

The comparison between groups of the main contributions of magnocellular and parvocellular systems to the VEP was further analyzed through measures of the corresponding nonlinearities expressed as neural efficiencies (ratios of second order nonlinear amplitude moderated by first order peak amplitudes). The results present a strong argument for enhanced parvocellular efficiency in the ASD group as measured by the reduction in the ratio of second to first order parvocellularly generated wave amplitudes.

Correlational analysis demonstrated relationships between global IT, parvocellular efficiency and Raven’s score in the ASD but not TD groups. The first relationship makes sense, when it is realized that the parvocellular efficiency is really a measure of the readiness of parvocellular neurons to fire after stimulation. Greater temporal readiness on the part of neurons is likely to enhance rapid recognition embodied in a short global IT. What stands out in the global IT data (which also showed a strong correlation with Raven’s score) is that group global IT performance for children who scored 30 and over on the RCPM test is not significantly different between ASD and Controls, yet the ASD group showed superior parvocellular efficiency. A more efficient parvocellular pathway could help explain why those with ASD are found to have enhanced local search skills (Shah and Frith, 1983; Mottron et al., 2003; Caron et al., 2006; Muth et al., 2014).

We propose that the three-way relation between non-verbal intelligence, global processing speed and parvocellular neural efficiency seen in the ASD but not the TD population suggests some sort of restriction on functional connectivity. While it is generally accepted that in the normal population there is a relation between non-verbal intelligence and visual processing speed as represented by IT (Nettelbeck, 1982; Barbeau et al., 2013), the mechanism of rapid processing would be attributed by most researchers to magnocellular function, certainly in terms of its role in figure-ground segregation (Bullier, 2001; Supér and Lamme, 2007). A disengagement of transient attention in ASD has been related to ineffectiveness of magnocellular/dorsal function (Laycock et al., 2007; Greenaway et al., 2013). To support this, our data (Figure 1) showed, for those in the ASD group, pattern recognition and hence global IT tend to be better in those with faster processing and neural recovery through the parvocellular system. However, it does not explain why local IT was not related in the same way. Our results support Van der Hallen et al. (2015) meta-analysis that found a difference in temporal pattern for ASD global/local processing.

We suggest that the three-way relation evident in our ASD group data are consistent with a constraint enforced by an ineffective magnocellular/dorsal network causing rapid recognition to be more reliant on restricted neural pathway connections to ventral stream processing. These connections are likely to be more dependent on parvocellular processing. Under such conditions, the relation between non-verbal intelligence and rapid neural processing would remain in ASD, but such rapidity would be more dependent on efficient parvocellular connections. The high discriminant accuracy of group prediction (ASD vs. TD) found in our data suggests utility in autism diagnosis, although further testing is required to assess the reliability of the findings.

## Author Contributions

DPC supervised the study. DPC and ACB were involved in the intellectual contributions for this study; analyzed the data; wrote the manuscript. ACB recruited the participants and ran the testing sessions with participants.

## Conflict of Interest Statement

The authors declare that the research was conducted in the absence of any commercial or financial relationships that could be construed as a potential conflict of interest.

## Footnotes

^1^www.vpixx.com
